# Effect of Er:YAG Pulsed Laser-Deposited Hydroxyapatite Film on Titanium Implants on M2 Macrophage Polarization In Vitro and Osteogenesis In Vivo

**DOI:** 10.3390/ijms25010349

**Published:** 2023-12-26

**Authors:** Lin Ma, Min Li, Satoshi Komasa, Shigeki Hontsu, Yoshiya Hashimoto, Joji Okazaki, Kenji Maekawa

**Affiliations:** 1 Department of Removable Prosthodontics and Occlusion, Osaka Dental University, 8-1 Kuzuhahanazono-cho, Hirakata 573-1121, Japan; ma-lin0617@outlook.com (L.M.); liminmin@outlook.jp (M.L.); 0100450@gmail.com (J.O.); maekawa-k@cc.osaka-dent.ac.jp (K.M.); 2 Department of Biomedical Engineering, Faculty of Biology-Oriented Science and Technology, Kindai University, 930 Nishimitani, Kinokawa 649-6493, Japan; hontsu@waka.kindai.ac.jp; 3 Department of Biomaterials, Osaka Dental University, 8-1 Kuzuhahanazono-cho, Hirakata 573-1121, Japan; yoshiya@cc.osaka-dent.ac.jp

**Keywords:** Er:YAG laser, pulsed-laser deposition, hydroxyapatite film, titanium implant, M2 macrophage polarization, osteogenic activity, osseointegration

## Abstract

In a previous study, we successfully coated hydroxyapatite (HAp) onto titanium (Ti) plates using the erbium-doped yttrium aluminum garnet pulsed-laser deposition (Er:YAG-PLD) method. In this study, we performed further experiments to validate the in vitro osteogenic properties, macrophage polarization, and in vivo osseointegration activity of HAp-coated Ti (HAp-Ti) plates and screws. Briefly, we coated a HAp film onto the surfaces of Ti plates and screws via Er:YAG-PLD. The surface morphological, elemental, and crystallographic analyses confirmed the successful surface coating. The macrophage polarization and osteogenic induction were evaluated in macrophages and rat bone marrow mesenchymal stem cells, and the in vivo osteogenic properties were studied. The results showed that needle-shaped nano-HAp promoted the early expression of osteogenic and immunogenic genes in the macrophages and induced excellent M2 polarization properties. The calcium deposition and osteocalcin production were significantly higher in the HAp-Ti than in the uncoated Ti. The implantation into rat femurs revealed that the HAp-coated materials had superior osteoinductive and osseointegration activities compared with the Ti, as assessed by microcomputed tomography and histology. Thus, HAp film on sandblasted Ti plates and screws via Er:YAG-PLD enhances hard-tissue differentiation, macrophage polarization, and new bone formation in tissues surrounding implants both in vitro and in vivo.

## 1. Introduction

Given the continuous innovations and developments in implant design and technology, implant prostheses have gradually become major treatment options for missing teeth [1,2]. The surface of a dental implant plays a significant role in the early stage of osseointegration, which directly affects the later stages of bone growth [3,4]. Osseointegration, which is defined as intimate contact between a new bone and the implant surface that occurs without the intervention of fibrous connective tissues, is an important aspect in improving the long-term clinical success of dental implants [5,6]. 

As the most widely used materials for dental implants, titanium (Ti) and its alloys can enhance bone bonding and prevent peri-implant bone resorption by modifying the implant surface [7,8]. The physical (e.g., roughness or topography) [7,9,10], chemical (e.g., coating) [11,12,13,14], and biochemical modification [15,16,17,18] of Ti implants changes the morphology, chemical composition, roughness, and hydrophilic properties of the implant surface, thereby improving the biocompatibility and bioactivity of the implant and facilitating the onset of osseointegration [18,19,20,21,22].

Nano-hydroxyapatite (HAp) is an appealing material for the deposition of bone implant coatings [23,24]. From a biomaterial perspective, thin-film HAp coatings exhibit inherently superior physical and chemical properties compared with other types of coating [25]. Furthermore, HAp is a major component of the hard tissues of living species, such as bones and teeth. It is chemically linked to tissues in the body, exhibits a certain level of solubility, and participates in biological metabolic processes to stimulate or produce bone hyperplasia and enhance bone-defect tissue healing in vivo [25,26,27,28,29,30]. Owing to its multisite binding properties with proteins, large specific surface area, high biocompatibility, bone inductivity, and other properties similar to those of natural bone, HAp is widely used in biomedical engineering as a bone scaffold, bone filler, implant coating, and drug-delivery system [31,32]. 

In a previous study, we successfully synthesized a HAp film on the surface of Ti substrates using the erbium-doped yttrium aluminum garnet pulsed-laser deposition (Er:YAG-PLD) method [33] and observed clear cellular responses, particularly enhanced osteogenesis. The next step in our work is to evaluate osteogenic efficacy using in vivo animal models to assess the practical applications of the coated materials.

Moreover, local trauma during the implantation process induces immune cells, such as macrophages and T cells, to congregate around the implant, owing to the immune reaction. Macrophages have been suggested as the main defense components of the innate immune system, which also includes dendritic cells, granulocytes, mast cells, and natural killer cells [34,35]. Macrophages secrete cytokines, chemical factors, and other substances that direct the recruitment, proliferation, and differentiation of bone-marrow-derived mesenchymal stem cells (BMMSCs) in response to local immune inflammatory reactions. After connecting to the implant surface, macrophages develop into M1 and M2 cells, depending on the surface features [36,37]. Specifically, M1 cells secrete interleukin 6 (IL-6), tumor necrosis factor (TNF), and other proinflammatory cytokines, whereas M2 cells secrete interleukin 10 (IL-10), vascular endothelial growth factor (VEGF), and other pro-tissue repair factors [38,39] to promote local inflammation. Because of their remarkable functional plasticity and ability to polarize between traditionally activated/inflammatory (M1) and alternatively activated/reparative (M2) phenotypes, macrophages have recently been shown to play a role in healing and tissue remodeling following injury [40]. The M1/M2 balance determines the ability of macrophages to play both positive and negative roles in disease processes and tissue healing [41]. Therefore, the identification of the mechanisms behind the functional polarization of macrophages into M1 or M2 cells could serve as a foundation for the development of macrophage-centered diagnostic and therapeutic techniques for a variety of disorders. Novel biomaterials are being developed to selectively polarize macrophages to obtain suitable responses. The physicochemical properties of implants, such as their surface topography, rigidity, and electrical properties, have been proposed to stimulate immune cells and regulate biological functions, indicating a promising approach to manipulating immunomodulation and to achieve favorable regeneration outcomes [42,43]. A significant amount of research has shown that changes in surface topography may potentially induce beneficial immunomodulation effects to enhance immune cell function in vitro and tissue creation in vivo [42,43,44,45,46,47]. Chen et al. reported that advances in micro and nanofabrication technologies have facilitated the creation of a variety of well-defined micro or nanotopographies on biomaterials and that topographies of varying scales appear to have different immunomodulatory effects [48].

Based on the above research, we believe that nano-HAp can strongly promote macrophage polarization in vitro and osteogenesis in vivo. Therefore, in this study, we aimed to evaluate implant surface bioactivity and osseointegration after HAp film modification. Specifically, we prepared a HAp film on Ti plates and screws using the Er:YAG PLD method and then examined the in vitro osteogenic properties, the macrophage polarization, the level of immune response from the macrophages, and the interaction between the macrophages and BMMSCs to enhance osseointegration. Furthermore, regarding the in vivo experiment, the osseointegration activity of the implant surface was also analyzed. 

## 2. Results

### 2.1. Microstructure of the Materials

#### 2.1.1. Surface Morphology

**Figure 1:**
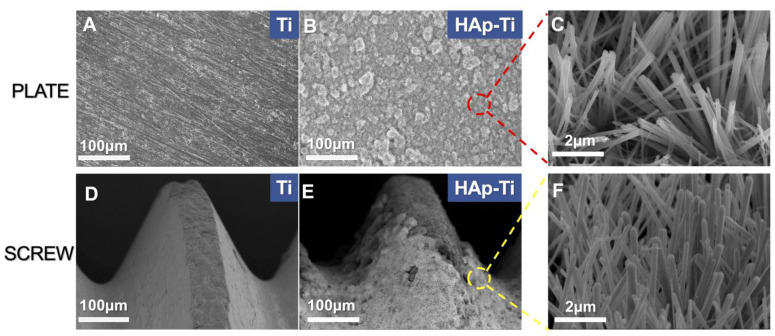
Surface microstructures of the samples at different magnifications. (**A**) Titanium (Ti) and (**B**) hydroxyapatite-coated Ti (HAp-Ti) plates. (**C**) Magnification of the area marked by the red circle in (**B**). (**D**) Ti and (**E**) HAp-Ti screws. (**F**) Magnification of the area marked by the yellow circle in (**E**).

Scanning-electron microscopy (SEM; S-4800; Hitachi, Tokyo, Japan) was used to investigate the surface characteristics of the pure titanium plates (Ti) and hydroxyapatite (HAp)-Ti. Figure 1A,B shows the surface microstructures of the (A) Ti and (B) HAp-Ti plates at the same magnification. Figure 1C presents the magnification of the area marked by the red rectangle in Figure 1B, which shows needle-like crystalline structures on the HAp-Ti plate. Figure 1D,E shows the micromorphology of the Ti screw and the HAp-Ti screw, and Figure 1F depicts the magnification of the area marked by the yellow rectangle in Figure 1E, the same needle-like crystalline structures as those found on the HAp-Ti screw.

#### 2.1.2. Compositional Analysis 

The microstructure and compositional distribution of the nanocrystals were investigated using energy dispersive X-ray spectroscopy (EDX) and X-ray photoelectron spectroscopy (XPS). 

**Figure 2:**
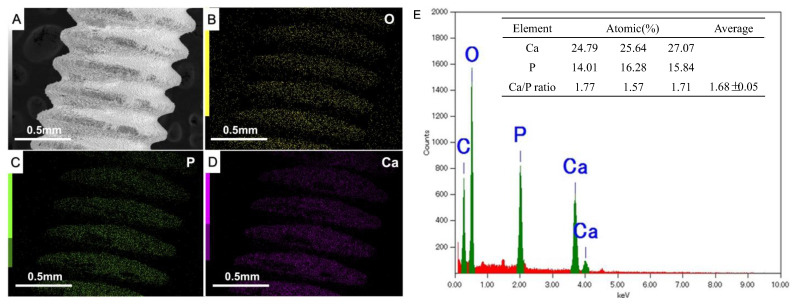
(**A**–**D**) Elemental mapping results in different magnifications of HAp-Ti screws; (**E**) energy-dispersive X-ray spectroscopy image of elemental composition of C (carbon) the calcium (Ca), phosphorus (P), and oxygen (O) of HAp-Ti screws.

An overlay of the calcium (Ca), phosphorus (P), and oxygen (O) EDX maps is shown in Figure 2. The elemental mapping results of this analysis confirmed a consistent distribution of O, P, and Ca in the screws at different magnifications, with an average Ca-to-P (Ca:P) ratio of 1.68, which is close to the average Ca:P ratio of stoichiometric HAp (1.67). The HAp film deposited on the implant surface was uniform and complete.

**Figure 3:**
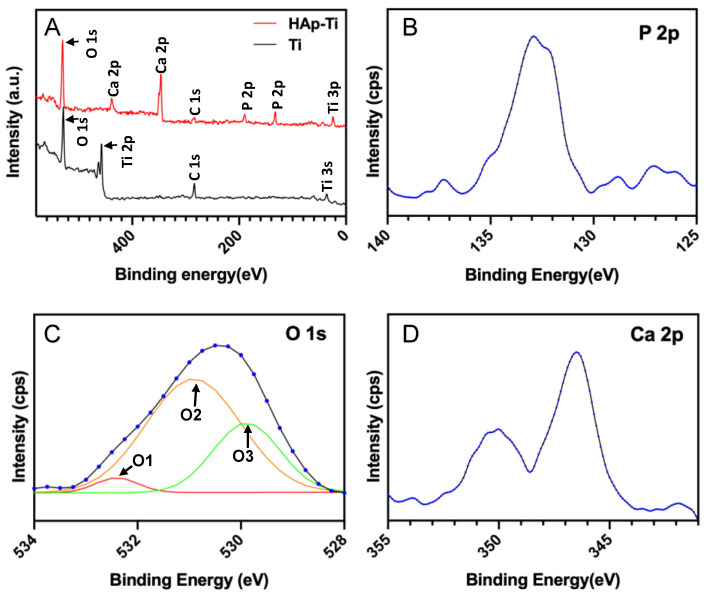
(**A**) Wide-spectrum X-ray photoelectron spectra of the HAp-Ti and Ti samples. (**B**–**D**) High-resolution (**B**) P 2p, (**C**) O 1s, and (**D**) Ca 2p XPS profiles of the HAp-Ti samples, the blue dotted lines represent fitted peaks, and peaks of different colors represent different chemical bonds.

The wide-scan XPS spectrum of the samples is shown in Figure 3. Figure 3A shows the presence of peaks attributable to Ca, O, and P, which are elements specific to HAp. This result clearly indicates that HAp was deposited on the Ti surface. The high-resolution P 2p, O 1s, and Ca 2p XPS profiles are presented in Figure 3B–D, respectively. The bonding energies corresponding to P 2p, O 1s, and Ca 2p were 132.9, 530.4, and 346.2 eV, respectively. The deconvolution of the O 1s peaks of all the samples revealed three O atomic states: the O1 peak (532.5 eV) corresponding to O^2−^ in the TiO_2_ lattice structure, the O2 peak (531.4 eV) corresponding to the O in –OH groups, and the O3 peak (529.8 eV) corresponding to the O in H_2_O.

#### 2.1.3. Crystalline Phase Identification 

**Figure 4:**
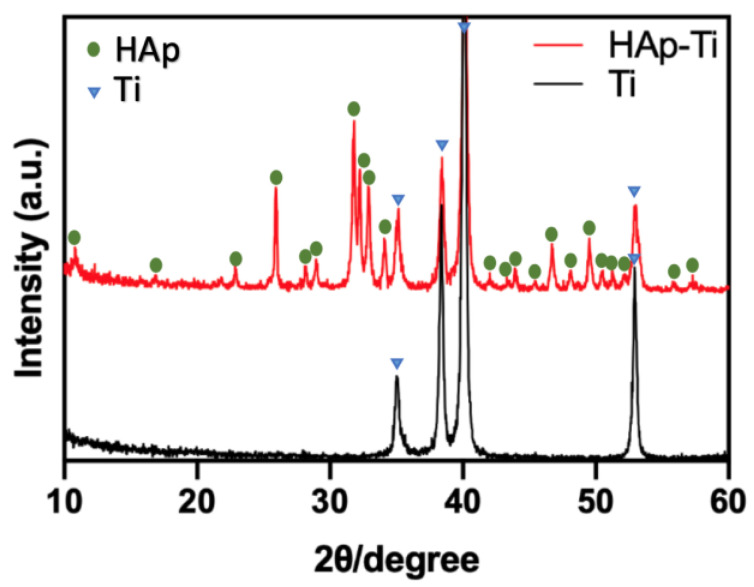
X-ray diffraction patterns of Ti and HAp-Ti.

The X-ray diffraction (XRD) patterns of the Ti and HAp-Ti are shown in Figure 4. The diffractograms show the characteristic peaks of the HAp, which are consistent with those of the standard HAp (JCPDS No. 72.1243), indicating that the crystalline structure of the HAp was maintained following its deposition as a thin film on the Ti surface.

### 2.2. In Vitro Experiments

#### 2.2.1. Cell Morphology of Macrophages

**Figure 5:**
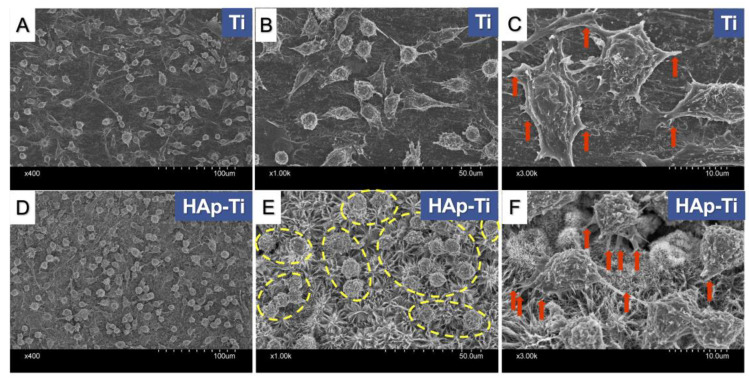
Morphology of macrophages on the (**A**–**C**) Ti and (**D**–**F**) HAp-Ti samples. The yellow circles indicate the macrophage population growing on HAp-Ti (**E**). Expanded magnification reveals clear cell morphology and cell pseudopods (marked by red arrows) growing on the surface of the samples (**C**,**F**).

Figure 5 shows the SEM images of the tested macrophages. The cell morphology was intuitively observed, and the cells exhibited obvious characteristics between the groups. Compared with those that grew on the Ti surface, the macrophages grown on the HAp-Ti surface had a flatter and broader spindle shape, as well as an expanded coverage area of all the cells on the material surface. The yellow circle indicates the macrophage population growing on the HAp-Ti (Figure 5E). The expanded magnification reveals clear cell morphologies and cell pseudopods (marked by red arrows) growing on the surfaces of the samples (Figure 5C,F).

#### 2.2.2. Polarization of Macrophages

**Figure 6:**
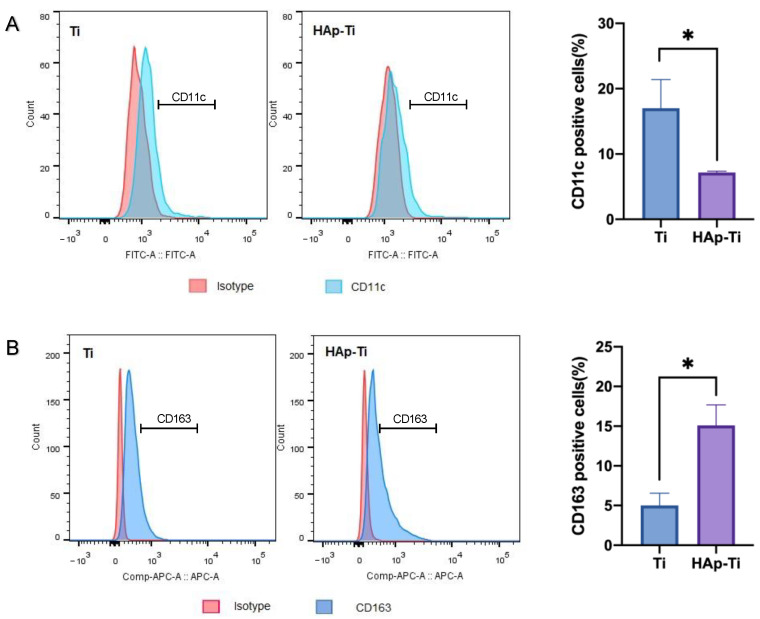
Fluorescence intensity of the (**A**) M1-related surface marker CD11c and (**B**) M2-related surface marker CD163 on the Ti and HAp-Ti, as assessed by flow cytometry (*n* = 4; * *p* < 0.05).

Figure 6A shows the fluorescence intensity of the M1-related surface marker CD11c, which was assessed using flow cytometry to evaluate the immunoregulatory effects of nano-HAp on macrophage polarization. The mean fluorescence intensity of the CD11c was reduced in the HAp-Ti relative to the Ti, indicating that the nano-HAp may have suppressed the proinflammatory reactions of the Ti. Conversely, Figure 6B shows the fluorescence intensity of the M2-related surface marker, CD163. The mean fluorescence intensity of the CD163 was increased in the HAp-Ti relative to the Ti, indicating that the nano-HAp may have improved the prohealing reactions of the Ti. 

#### 2.2.3. Osteogenic Activity of Macrophages

**Figure 7:**
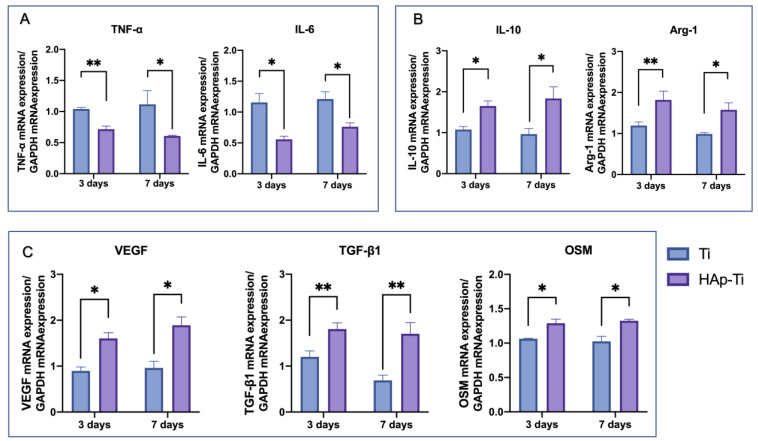
(**A**) Expression of the proinflammatory genes TNF-α and IL-6. (**B**) Expression of the anti-inflammatory genes IL-10 and Arg-1. (**C**) The mRNA expression of angiogenesis-, fibrogenesis-, and osteogenesis-related genes (*n* = 4; * *p* < 0.05, ** *p* < 0.01).

After incubating the macrophages for 3 and 7 d, the expression of the M1- and M2-related genes was examined using a quantitative real-time polymerase chain reaction (RT-qPCR). As shown in Figure 7, the expression of proinflammatory genes, such as (A) TNF-α and IL-6, was dramatically downregulated by the HAp-Ti, whereas the expression of anti-inflammatory genes, such as (B) IL-10 and Arg1, was significantly elevated. These findings indicate that nano-HAp reduces the degree of the proinflammatory response in macrophages.

The mRNA expression of (C) angiogenesis-, fibrogenesis-, and osteogenesis-related genes, all of which play important roles in the production of new bone, was also investigated. The macrophages grown on the HAp-Ti showed higher gene expression levels than those grown on the Ti. 

#### 2.2.4. Osteogenic Activity of Rat-Bone-Marrow-Derived Stem Cells (rBMMSCs)

**Figure 8:**
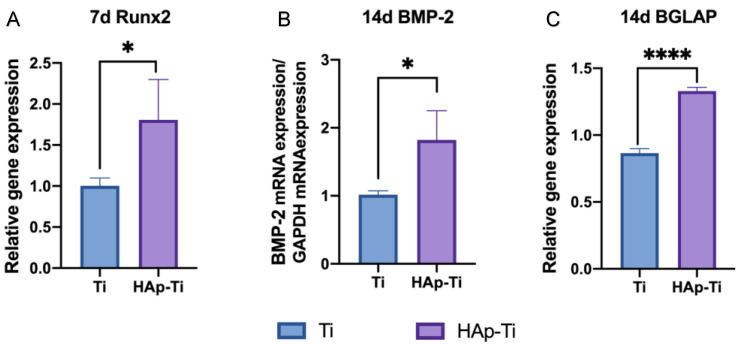
The mRNA expression levels of (**A**) Runx2 after 7 d and (**B**) BMP-2 and (**C**) BGLAP after 14 d (*n* = 4; * *p* < 0.05, **** *p* < 0.0001).

The gene expression related to the induction of hard-tissue differentiation was analyzed. The mRNA expression levels of osteogenesis-related genes, including runt-related transcription factor (Runx2), bone morphogenetic protein 2 (BMP-2), and bone carboxyglutamate protein (BGLAP), were assessed using a TaqMan quantitative real-time polymerase chain reaction (PCR). As shown in Figure 8, compared with the Ti, the HAp-Ti showed higher levels of Runx2 mRNA expression after 7 d of culture and higher levels of BMP-2 and BGLAP mRNA expression after 14 d of culture.

**Figure 9:**
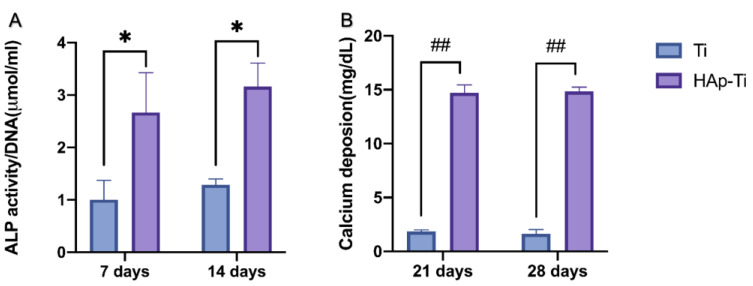
Quantitative results of (**A**) ALP activity after 7 and 14 d and (**B**) Ca deposition after 21 and 28 d (*n* = 4; * *p* < 0.05, ^##^ *p* < 0.000001).

Figure 9 shows the quantitative results of ALP activity after 7 and 14 d. The relative ALP activity of the cells on the HAp-Ti samples was significantly higher than that of the cells on the Ti sample. In each group, the Ca deposition, a late marker of extracellular matrix mineralization, was quantified after 21 and 28 d of differentiation. The HAp-Ti samples exhibited higher levels of Ca deposition than the Ti samples on days 21 and 28. 

#### 2.2.5. Osteogenic Differentiation of rBMMSC in Macrophage-Conditioned Medium

**Figure 10:**
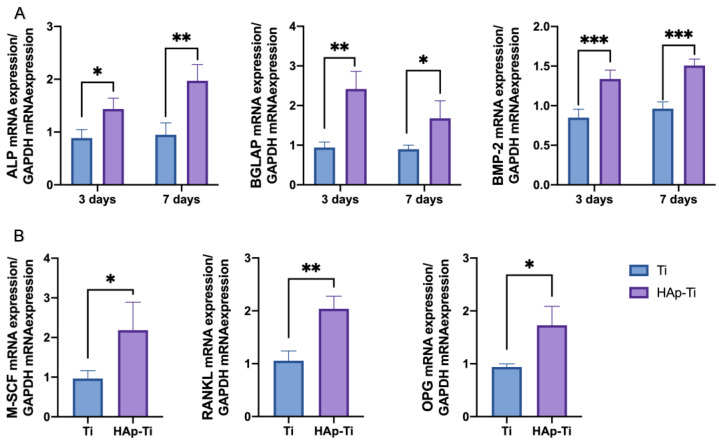
The mRNA expression levels of (**A**) ALP, BGLAP, and BMP2 after 3 d and 7 d and (**B**) M-SCF, RANKL, and OPG after 3 d (*n* = 4; * *p* < 0.05, ** *p* < 0.01, *** *p* < 0.001).

The expression of osteogenic genes (ALP, BMP-2, and BGLAP) in the rBMMSCs cultured with different CM for 3 and 7 days was determined using RT-qPCR, as shown in Figure 10A. The HAp-Ti groups demonstrated a high expression of all the genes at both time points, indicating that the HAp-Ti coculture system exhibited a higher capacity to promote the osteodifferentiation of rBMMSCs. Moreover, the expression of osteoclastogenesis-related genes (M-SCF, RANKL, and OPG) was significantly upregulated in the HAp-Ti cultured with different CM for 3 days in Figure 10B, which suggests enhanced osteoclastogenesis and osteogenic activities, further indicating that the needle-like hydroxyapatite might have induced an active bone-reconstruction process.

### 2.3. In Vivo Experiments

#### 2.3.1. Evaluation of Hard-Tissue Differentiation

**Figure 11:**
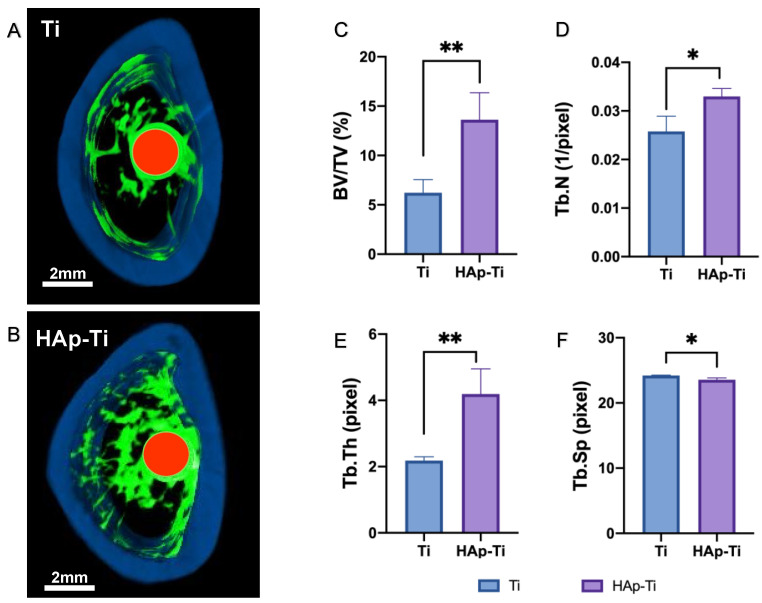
(**A**,**B**) Reconstructed three-dimensional microcomputed tomography (micro-CT) images of Ti and HAp-Ti samples (red, implants; green, cancellous bone; blue, cortical bone), (**C**) bone volume-to-total volume (BV/TV) ratio, (**D**) mean trabecular number (Tb.N), (**E**) mean trabecular thickness (Tb.Th), and (**F**) mean trabecular separation (Tb.Sp) of the implants (*n* = 8; * *p* < 0.05, ** *p* < 0.01).

A rat femur model was selected to assess the osteogenic activity around the implants in each group. The reconstructed three-dimensional microcomputed tomography (micro-CT) images of the implants are shown in Figure 11A,B. More trabecular microstructures were observed around the HAp-Ti implant surface than around the Ti surface after 8 weeks. The new bone formation around the implants was greater in the HAp-Ti group than in the Ti group. Additionally, the HAp-Ti samples showed a higher bone volume-to-total volume (BV/TV) ratio, mean trabecular number (Tb.N), and mean trabecular thickness (Tb.Th) than the Ti samples. The mean trabecular separation (Tb.Sp) was higher in the HAp-Ti implants than in the Ti implants (Figure 11C–F).

#### 2.3.2. Quantification of New Bone Formation

**Figure 12:**
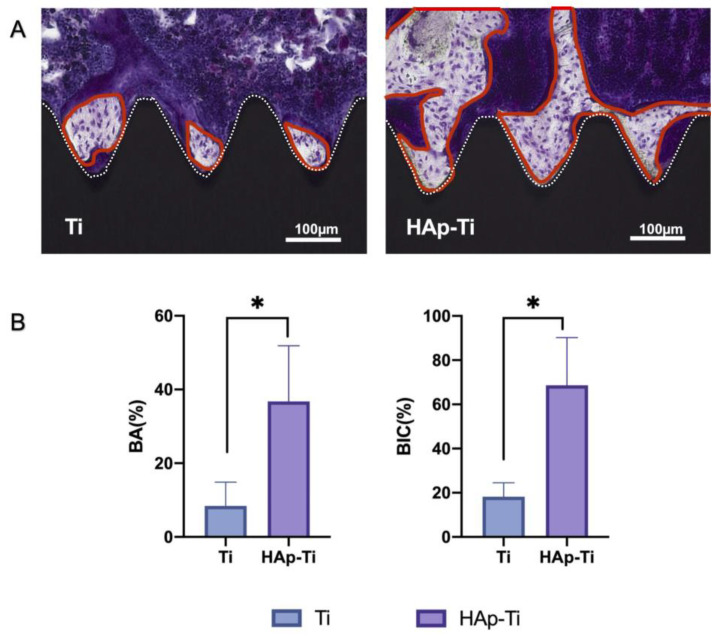
(**A**) Images of the longitudinal sections of the implants and surrounding bone tissues. (**B**) Histomorphometric analysis of the bone area (BA) ratio and bone-to-implant contact (BIC) around the HAp-Ti and Ti implants (*n* = 8; * *p* < 0.05).

Images of the longitudinal sections of the implants and surrounding bone tissues are presented in Figure 12A. The areas circled in red represent new bone, and the white lines indicate the site of contact between the Ti screws and bone tissues. Compact, adherent, new bone tissue was visible on the implant surfaces. However, more new bone cells were observed around the surfaces of the HAp-Ti implants than around the surfaces of the Ti implants, as shown in Figure 12A. The histomorphometric analysis showed that the bone area ratio (BA) and bone-to-implant contact (BIC) were higher around the HAp-Ti implants than around the Ti implants (Figure 12B).

## 3. Discussion

In this study, the HAp was coated onto the Ti plates and screws via Er:YAG-PLD. We then validated in vitro the osteogenic properties and macrophage polarization, as well as the in vivo osseointegration activity of the coated samples. Compared with those in the Ti group, the macrophages grown on the surface of the HAp-Ti differentiated into M2 cells quickly and promoted the repair of the trauma tissue around the HAp-Ti. Moreover, compared with the Ti group, the HAp-Ti group revealed larger amounts of new bone formation in the surrounding tissues. These results indicate that the Er:YAG-PLD approach is a suitable implant surface modification technology that could potentially be applied in the clinical setting.

Previous studies revealed that Er:YAG-PLD can be used to coat α-TCP, β-TCP, HAp, and other materials onto Ti plates [26,49,50,51,52,53]. Our previous studies demonstrated that HAp can be coated onto Ti plates via Er:YAG-PLD [33,51,52,53]. In the present study, α-TCP powder was selected as the target because α-TCP particles are smaller than HAp particles; thus, when the powder is sprayed on the Ti surface after laser irradiation, a dense α-TCP film is formed. Correspondingly, when this film is hydrated into a HAp film, the density of the resulting film is also high, and the performance and bond strength of the film are enhanced. These features improve aspects of the performance of the film, such as its ability to bond with other materials and the rate at which it releases compounds in the body [54]. Sandblasting pretreatment is often performed on Ti flakes because sandblasting has a beneficial effect on osteogenesis, both in vitro and in vivo. Sandblasting is a surface treatment used to improve the surface characteristics of Ti [55]. Because Er:YAG-PLD is performed manually at the chairside, it cannot guarantee film homogeneity [49]. Therefore, Ti-sandblasting pretreatment may play an important role in enhancing the surface characteristics of an implant, even in areas that cannot be covered by HAp, and surfaces where hydroxyapatite has been resorbed, so that the implant is exposed directly to the bone, can have excellent osteogenic results.

In this study, we coated an α-TCP thin film onto Ti plates and screws via Er:YAG-PLD and then immersed the coated materials in ultrapure water at 90° for 10 h to hydrate and crystallize the HAp. According to the low-magnification SEM results, the HAp-Ti plate and screw samples exhibited a nanorod-like morphology. The XPS detected Ca, P, and O. The O1s results showed the presence of –OH, Ti–O, and H_2_O groups in the HAp-Ti samples. This finding implies that a connection existed between the thin film and the Ti surface. The presence of –OH and H_2_O, which are hydrophilic functional groups, may facilitate cells’ attachment to the material surface [9]. The EDS also detected Ca, P, and O, and the Ca:P ratio of the HAp-Ti samples was 1.68, which is nearly the average Ca:P ratio in stoichiometric HAp (1.67). The difference between the Ca:P ratios of the HAp-Ti and the original α-TCP (nearly 1.5) may be attributed to the fact that during the hydrothermal reaction, the Ca^2+^ and PO_3_^4−^ dissolved into the water and combined with the OH^−^ and H^+^ to produce HAp; differences in the release rates of Ca^2+^ and PO_3_^4−^ may have led to the observed difference between the Ca:P ratios. Moreover, the XRD results indicated that the crystalline structure of the HAp was preferentially oriented along the C axis and demonstrated peaks that were consistent with those in the standard card of HAp; this finding confirmed that HAp was successfully prepared on the Ti [56]. Taken together, the surface morphological, elemental, and crystallographic analyses results confirmed the successful deposition of a HAp film on the surfaces of the Ti plates and screws. 

**Figure 13:**
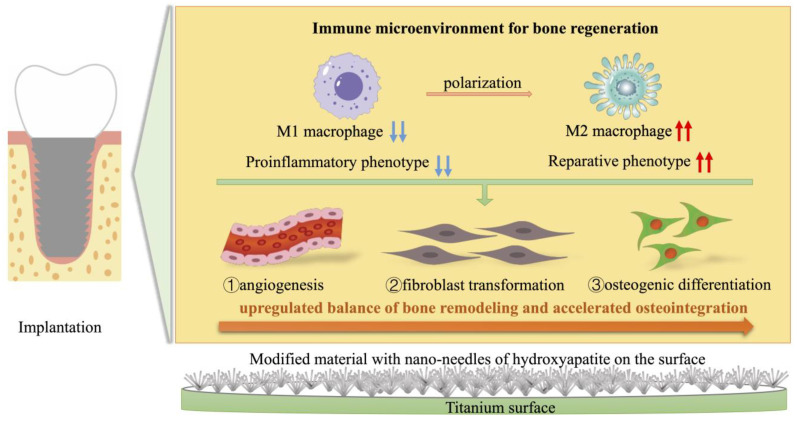
Schematic illustration of the immunomodulation strategy based on modified material with nano-needles of hydroxyapatite on the implant surface (the rat femur was used as an implant model in this experiment, the blue downward arrows represent negative effects and the red upward arrows represent positive effects).

Immune cells and rBMMSCs are involved in bone-graft implantation, and macrophages may be among the first cell types to appear in the areas surrounding an implant [57]. Thus, macrophage activation and its influence on osteogenesis are critical for understanding the interactions of bone-implant biomaterials with the bone-defect microenvironment. We utilized RAW264.7 cells to study the various behavioral and biological responses of immune cells to the Ti and HAp-Ti samples. The SEM showed that the HAp-Ti had more macrophages than the Ti. In addition, the cells were more firmly stretched and uniformly dispersed on the HAp-Ti surface than on the Ti surface. The fluorescence intensity of the M1-related surface marker, CD11c, and the M2-related surface marker, CD163, the mean fluorescence intensity of the CD11c was reduced and the CD163 was increased in the HAp-Ti relative to the Ti, indicating that the nano-HAp may have suppressed the proinflammatory reactions and improved the prohealing reactions of the Ti. The expression of inflammation-related genes (IL-6 and TNF-α) in the macrophages in the HAp-Ti group showed significant reductions compared with those in the Ti group, whereas the expression of the genes involved in the M2 macrophage differentiation (IL-10 and Arg-1) was greater on the HAp-Ti than on the Ti. This result indicates that HAp-Ti may direct macrophage differentiation into the M2 phenotype while promoting tissue repair and healing. Furthermore, TNF-α and IL-6 can control cell death in inflamed tissues, alter vascular endothelial permeability, and stimulate C-reactive protein synthesis in the acute phase. Along with other inflammatory cytokines, TNF-α induces the transitory activation of TNF-α and Janus kinase-signal transducers, as well as the activators of transcription (JAK-STAT) signaling pathways, which contribute to the increased expression of transcription factors such as NF-B and RANKL/RANL/OPG [58,59,60,61,62]. According to the gene-expression data, the macrophages cultured on the HAp-Ti showed higher levels of angiogenesis (VEGF), fibroblast transformation (TGF-β1), and osteogenic differentiation (OSM), as shown in Figure 7. A specific diagram of the experimental mechanism can be found in Figure 13. Yang et al. demonstrated that different structures can influence different osteoimmune environments to improve subsequent osteogenesis, and that micro/nano hierarchical structures have the potential to promote better bone regeneration by generating a favorable immune environment for osteogenesis [48,63]. These findings are consistent with the experimental results of the present study.

The ALP activity and Ca deposition results indicated that the rBMMSCs [58] on the HAp-Ti had a higher level of differentiation ability for osteogenesis than those on the Ti. These findings confirm that HAp and sandblasting positively affect initial osteogenic differentiation in vitro. Furthermore, the RT-qPCR revealed that the expression levels of Runx2, BMP-2, and BGLAP on the HAp-Ti were extremely high. The transcription factor, Runx2, is abundantly expressed in the early stages of osteogenesis, whereas BMP-2 and BGLAP are expressed in its advanced stages. These results imply that the HAp film significantly enhances osteogenesis and biocompatibility while retaining the properties of HAp. 

Regarding the positive impact of HAp-Ti on bone formation in bone marrow mesenchymal cells, it is not sufficient to demonstrate the influences by only facilitating macrophage differentiation into M2 cells. This is better examined the consequences of cellular interactions between macrophages and bone marrow mesenchymal stem cells [64,65,66,67]. Consequently, to replicate the bone marrow milieu surrounding the implant, we cultivated bone marrow mesenchymal stem cells using a conditioned medium that included macrophage secretion, and then examined the osteogenic (ALP, BMP-2, and BGLAP) and osteoblastic (M-CSF, CSF1, and OPG) gene expression in the bone marrow mesenchymal stem cells within the conditioned media. The M-CSF, also known as colony-stimulating factor 1 (CSF1), is a dimeric glycoprotein secreted by osteoblasts and linked by interchain disulfide bonds. It is a hematopoietic growth factor involved in the proliferation, differentiation, and survival of monocytes, macrophages, and bone marrow progenitor cells, and its local production in the vessel wall contributes to the process of atherosclerosis. Some related studies have shown that M-CSF and vascular endothelial growth factor (VEGF) can induce osteoclastogenesis. Osteoclast differentiation, maturation, and function are induced by the binding of RANKL expressed by osteoblasts, osteocytes, and stromal cells to RANK on osteoclasts. Osteoprotegerin (OPG) is an inhibitor of osteoclast differentiation that is produced by a variety of cells, including osteoblasts, fibroblasts, and hepatocytes. It inhibits RANKL-mediated signaling by binding to RANK in osteoclasts. Regarding the osteogenesis-related gene expression of bone marrow mesenchymal stem cells, the conditioned medium obtained from the macrophages cultured on the HAp-Ti exhibited a stronger effect on the promotion of bone remoding effect compared to the medium obtained from the macrophages cultured on the Ti. Thus, HAp-Ti implants can direct macrophages to generate more osteogenic cytokines that promote osteogenic differentiation, thus accelerating the osteogenic process and resulting in high bone mass and quality after implantation.

In the in vivo experiments, more trabecular microstructures were observed around the HAp-Ti implant surface than around the Ti surface after 8 weeks. The new bone formation around the implants was greater in the Hap-Ti group than in the Ti group. Additionally, the Hap-Ti samples showed a higher BV/TV ratio, Tb.N, Tb.Th, and Tb.Sp than the Ti samples. These results indicate that Hap promotes osteoblast growth and induces in vitro bone formation, as well as osteoblast proliferation and differentiation, which is consistent with the results of previous studies [63,64,65,66,67,68,69,70,71,72,73,74,75]. According to the in vivo experiments, the surfaces of the HAp-Ti implants showed widespread new bone and tissue production. Thus, we hypothesize that HAp-Ti can increase osseointegration in Ti implants, which would improve their long-term durability.

Although important discoveries were revealed in this study, some limitations remain. For instance, we did not determine the stability or release rate of the deposited film. Moreover, we did not assess the homogeneity of the film. Studies related to these topics can be performed to better characterize the properties of HAp-Ti.

## 4. Materials and Methods

### 4.1. Sample Preparation

The Ti plates measuring Φ15 mm × 1 mm were polished for 1 min using SiC abrasive papers (#800 and #1200), followed by an MG400-CS micro-grinding machine (Meiwafosis, Tokyo, Japan). The samples and Ti screws measuring Φ1.2 mm × 12 mm were ultrasonically cleaned with acetone, ethanol, and deionized water in sequence for 10 min each time and used as the Ti control. 

### 4.2. Fabrication of the HAp-Ti Plate and Screw

**Figure 14:**
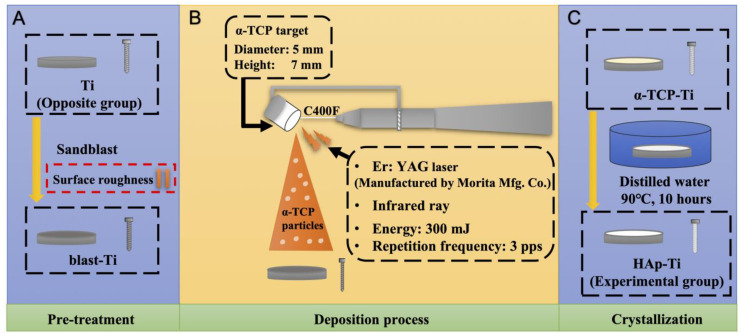
Fabrication process of the HAp-Ti plate and screw. (**A**) The pre-treatment of the experiment. (**B**) The deposition process of the experiment. (**C**) The crystallization of the experiment.

The Ti plates were sandblasted (blast-Ti) as a pretreatment to increase their surface roughness. The α-TCP (Taihei Chemical Industrial, Osaka, Japan) was then deposited on blast-Ti using an Er:YAG laser unit (Erwin AdvErl Unit; Morita Manufacturing, Kyoto, Japan) at a pulse energy of 300 mJ and frequency of 3 pps (α-TCP-Ti). The α-TCP-Ti was immersed in ultrapure water at 90 °C for 10 h to hydrolyze the film surface and obtain HAp (HAp-Ti). A schematic of the fabrication process is given in Figure 14.

### 4.3. Surface Characterization

The surface morphologies of the Ti and HAp-Ti samples were observed by SEM (S-4800; Hitachi, Tokyo, Japan). The SEM samples were coated with a thin and conductive osmium (Os) layer using an Os coating machine (HPC-20; Vacuum Device, Ibaraki, Japan). We used XRD (Ultima IV, Rigaku, Tokyo, Japan) to determine the crystallinity of the films. The analysis was performed using Cu Kα radiation at 40 kV and 100 mA with a scan speed of 2°/min, an incidence angle of 1°, and a 2θ range of 10°–60°. We used EDX (JED-2300, JEOL, Tokyo, Japan) to determine the elemental composition of the samples.

### 4.4. In Vitro Experiments

#### 4.4.1. Cell Culture

Rat BMMSCs (rBMMSCs) (Shimizu Laboratory Supplies Co., Kyoto, Japan) and the murine-derived macrophage cell line (RAW264.7) (EC91062702, KAC Co., Kyoto, Japan) were used in this study. The rBMMSCs were obtained from the femurs of eight-week-old Sprague–Dawley rats and cultured in 75 cm^2^ flasks with Eagle’s minimum essential medium (E-MEM; Nacalai Tesque, Inc., Kyoto, Japan) containing 10% fetal bovine serum (FBS) and an antibiotic–antimycotic solution (all from Nacalai Tesque, Inc., Kyoto, Japan) at 37 °C according to the protocol described in a previous article [39]. Third-generation cells were used for the in vitro experiments. The cells were digested in a solution containing 0.5 g/L trypsin and 0.53 mmol/L EDTA (Nakalai Tesque Inc., Kyoto, Japan), centrifuged, resuspended, added to the disc samples, and placed in a 24-well plate at a density of 5 × 10^4^ cells/well. The medium was changed every 3 d. 

The murine macrophage cell line RAW264.7 (EC91062702; KAC Co., Kyoto, Japan) was used to evaluate the macrophage polarization in our research. The RAW264.7 cells were maintained in α-MEM, (Nacalai Tesque, Inc., Kyoto, Japan) supplemented with 10% FBS and 1% penicillin/streptomycin, and cultured at 37 °C with 5% CO_2_. Adherent cells were dislodged upon reaching approximately 80% confluence by gently passing a cell scraper over them. The cells were used for the in vitro experiments at a density of 10^5^ cells/well. Meanwhile, on days 3 and 7, the conditioned media were collected and centrifuged (1500× *g* rpm at 4 °C) to obtain the supernatants. The obtained supernatant was combined with the culture medium (MEM with 10% FBS and 1% penicillin/streptomycin, with or without osteogenic additives) at a 2:1 ratio to create the conditioned medium (CM) used for additional experiments. The Osaka Dental University criteria for animal testing were used in this study (Approval No. 21-09002).

#### 4.4.2. Polarization of Macrophages

Flow cytometry was used to assess the expression levels of the M1-cell-surface marker, CD11c, and the M2-cell-surface marker, CD163, to examine the effect of nano-HAp on macrophages. The RAW264.7 cells were seeded onto the Ti and HAp-Ti samples at a density of 10^5^ cells/well for 7 d and then gently scraped off. Nonspecific protein binding was inhibited with 1% BSA/PBS before staining with CD11c Monoclonal Antibody (eBio-scienceTM, Tokyo, Japan) and CD163 Monoclonal Antibody (eBio-scienceTM, Tokyo, Japan) for 30 min at 4 °C in the dark. Armenian Hamster IgG Isotype Control (eBio299Arm, Alexa Fluor 488, eBioscienceTM, Kyoto, Japan) and Armenian Hamster IgG2a Isotype Control (eBio299Arm, Alexa Fluor 488, eBioscienceTM, Kyoto, Japan) were employed as the isotype control. The cells were examined using a Beckman Coulter FC500 flow cytometer (Brea, CA, USA) after washing with 1% BSA/PBS. FlowJo X software (TreeStar Inc., Ashland, OR, USA) was used to analyze the data.

#### 4.4.3. Gene Expression of Inflammatory Macrophages

The RAW264.7 cells were seeded on the Ti and HAp-Ti samples in a 24-well plate at a density of 10^5^ cells/well. The cells were incubated for 7 d, and the medium was changed on day 3. On days 3 and 7, the conditioned media were collected and centrifuged (1500× *g* rpm at 4 °C) to obtain the supernatants. The culture medium (MEM containing 10% FBS and 1% penicillin/streptomycin, with or without osteogenic supplements) was mixed with the acquired supernatant at a ratio of 2:1 to obtain the conditioned medium (CM) for further experiments.

The expression levels of inflammation-related genes from the M1 phenotype (TNF-α, IL-6) and the M2 phenotype (IL-10, Arg-1) were evaluated using real-time TaqMan reverse transcriptase polymerase chain reaction (RT-qPCR) assay (Life Technologies, Carlsbad, CA, USA). The Ct method was used to determine the relative gene-expression levels in each group, which were normalized to that of the housekeeping gene, glyceraldehyde 3-phosphate dehydrogenase (GAPDH).

#### 4.4.4. Gene Expression of Osteogenic Macrophages

The expression levels of transforming growth factor beta 1 (TGFβ1), vascular endothelial growth factor (VEGF), and oncostatin M (OSM) were analyzed by RT-qPCR, as described in Section 4.4.5 to investigate the effects of HAp-Ti on osteogenic gene expression in RAW264.7 cells.

#### 4.4.5. Alkaline Phosphatase (ALP) Activity of rBMMSCs

After incubation for 7 d, the medium was replaced with the differentiation-inducing medium α-MEM (Nacalai Tesque Inc.) containing 10% FBS, an antibiotic–antimycotic mix, the osteogenic supplements 10 mM β-glycerophosphate (Wako Pure Chemical Industries Ltd., Kyoto, Japan), and 10 nM dexamethasone (Nacalai Tesque Inc.). The medium was changed every 3 d. After incubation for 7 or 14 d, the ALP activity of the rBMMSCs was measured using ALP pNPP Liquid Substrate and enzyme-linked immunosorbent assay (ELISA) kits (Sigma-Aldrich, St Louis, MO, USA). The DNA content of each sample was measured using the PicoGreen dsDNA Assay Kit (Thermo Fisher Scientific, Waltham, MA USA) according to the manufacturer’s protocol. The *p*-nitrophenol production was determined using a 96-well microplate reader (SpectraMax^®^ M5; Molecular Devices, San Jose, CA, USA) at 405 nm, and the amount of ALP was normalized against that of DNA in the cell lysates. 

#### 4.4.6. Extracellular Matrix Mineralization

Extracellular matrix mineralization in the rBMMSCs was detected using a Calcium E-Test Kit (Wako Pure Chemical Industries). After culturing for 28 d, 1 mL of the Calcium E-Test reagent and 2 mL of the kit buffer were mixed and added to the cells. The absorbance of the reaction solution was measured at 612 nm using a 96-well microplate reader (SpectraMax^®^ M5).

#### 4.4.7. Osteogenesis and Osteoclastogenesis-Related Gene Expression of rBMMSC

A macrophage-rBMMSC co-culture system was constructed utilizing the conditioned medium (CM) approach to explore whether RAW264.7 cells might influence the osteogenesis of rBMMSC. The RAW264.7 cells from Ti and HAp-Ti groups were collected and mixed with MEM as above. The rBMMSC cells were seeded at a density of 5 × 10^4^ cells/well in a 24-well plate and incubated at 37 °C for 24 h. After incubation, the culture medium was removed and replaced by CM. Bone-related genes, alkaline phosphatase (ALP), bone morphogenetic protein 2 (BMP-2), and bone carboxyglutamate (gla) protein (BGLAP), and osteoclastogenesis-related genes, OPG, RANKL, and MCS-F, were analyzed by RT-qPCR after 3 and 7 d of culture.

### 4.5. In Vivo Evaluation of Osteointegration

#### 4.5.1. Implantation into Rat Femurs

The animal procedures and experiments were performed in accordance with the ethical principles of the National Institutes of Health Guide for the Care and Use of Laboratory Animals and were approved by the Medical Ethics Committee of Osaka Dental University (approval no. 21-09002). Sixteen 8-week-old male Sprague–Dawley rats (Shimizu Laboratory Supplies Co., Tokyo, Japan), weighing 180–200 g each, were randomly divided into two groups and used in this study. After general anesthesia, the distal aspects of the femurs were carefully exposed via a 10 mm vertical skin incision at the knee joint. A 1.2 mm pilot hole was drilled into the distal femur, as described in previous studies [13,16,71]. The Ti and HAp-Ti screws were then randomly implanted into the bilateral femurs of the rats. The surgical sites were carefully closed in layers. 8 weeks after surgery, the rats were sacrificed via an intraperitoneal injection of sodium pentobarbital, and bilateral femurs containing implants were harvested. Gentamicin (1 mg/kg) and buprenorphine (0.05 mg/kg) were injected for 3 d after surgery to prevent postsurgical infection and decrease postoperative pain.

#### 4.5.2. Micro Computed Tomography Evaluation

A micro-CT system (SkyScan1275, Bruker, MA, USA) operated at 90 kV and 40 μA was applied to investigate the effects of HAp on the formation of new bone around the implants, which were taken after 8 weeks after surgery. The bone volume fraction (BV/TV), trabecular number (Tb.N), trabecular separation (Tb.Sp), and trabecular thickness (Tb.Th) within the regions of interest (ROI; 300 μm around the implant and 2 mm below the epiphyseal line) in the CT images were determined using Morphometric software CTAn version 1.19.11 (Bruker, Billerica, MA, USA). 

#### 4.5.3. Histological Evaluation 

After micro-CT scanning, the femoral specimens that were taken after 8 weeks after surgery were collected, dehydrated, and stained using the Villanueva method to observe bone formation under a BZ-9000 digital cold illumination microscope (Keyence Co., Osaka, Japan). The BA and BIC around the implants were assessed using ImageJ software (https://imagej.net (accessed on 1 December 2023)) at a magnification of 200×.

### 4.6. Statistical Analysis

All quantitative results are expressed as mean ± standard deviation. Statistical significance was analyzed using one-way analysis of variance (ANOVA) and Bonferroni’s post hoc test with GraphPad Prism 8.0 software (GraphPad Prism, San Diego, CA, USA). In this study, *p* < 0.05 was considered statistically significant, while *p* < 0.01 was considered highly significant. All experiments were conducted in triplicate. 

## 5. Conclusions

The results indicated that HAp can be easily deposited on Ti surfaces using an Er:YAG laser. The needle-shaped nano-HAp promoted the early expression of osteogenic and immunogenic genes in the macrophages and showed excellent M2 polarization properties. The Ca deposition and osteocalcin production were significantly higher in HAp-Ti than in the uncoated Ti. The implantation into the rat femurs also revealed that the HAp-coated materials had superior osteoinductive and osseointegration activities compared with the Ti, as assessed by the micro-CT and histology. The HAp film improved the induction of hard-tissue differentiation and the formation of new bone in the tissue surrounding the implant, both in vitro and in vivo. 

In conclusion, Er:YAG-PLD can be employed as a viable strategy for Ti-implant-surface modification with HAp films. This method was proven to be an excellent surface-treatment technology for oral implants. We plan to investigate additional HAp film characteristics in future work and to provide further data for its possible therapeutic applications.

## Data Availability

Data are contained within the article.
